# Midterm outcomes of single port thoracoscopic surgery for major pulmonary resection

**DOI:** 10.1371/journal.pone.0186857

**Published:** 2017-11-14

**Authors:** Kook Nam Han, Hyun Koo Kim, Young Ho Choi

**Affiliations:** 1 Department of Thoracic and Cardiovascular Surgery, Korea University Guro Hospital, Seoul, Republic of Korea; 2 Department of Thoracic and Cardiovascular Surgery, Korea University College of Medicine, Seoul, Republic of Korea; Baylor College of Medicine, UNITED STATES

## Abstract

**Introduction:**

Single-port thoracoscopic surgery has widened the current minimally invasive surgical techniques toward more less invasive procedures in terms of reducing the number of incisions. However, the current status of oncologic outcome with this technique is not well known for lung cancer surgery. The purpose of this study is to evaluate the oncologic outcomes in early stage lung cancer for impact of the survival outcomes with our experience of conversion to a single-port approach from the conventional three-port approach.

**Materials and methods:**

Retrospective data of patients who underwent thoracoscopic major lung resection for non-small cell lung cancer between January 2006 and June 2015 were analyzed. Patients’ characteristics, perioperative outcomes, pathologic result, and postoperative follow-up data of thoracoscopic surgery were reviewed and surgical outcomes were compared between conventional three-port (n = 168), two-port (n = 68), and single-port thoracoscopic surgery (n = 203).

**Results:**

Of the 203 single-port thoracoscopic surgeries, we performed 167 single-port thoracoscopic lobectomy and mediastinal lymph node dissections. During the learning period of each thoracoscopic approach, the mean operation time for single-port thoracoscopic surgery (189±62 min) was not significantly different from those of two-port (175±46 min) and three-port (195±75 min) thoracoscopic lobectomy (*p* = 0.165). Perioperative outcomes including drain indwelling time (*p* <0.001), complication (*p* = 0.185) and conversion event (p = 0.911) were not worsened during learning period with two-port. Midterm survival (*p* = 0.753) and recurrence free survival (*p* = 0.656) of single port thoracoscopic lobectomy showed acceptable results compared with two- and three-port approach.

**Conclusions:**

Single-port thoracoscopic surgery is safe and a feasible option for major lung resection in lung malignancy and this approach following experiences of two-port approach may yield similar oncologic results to those of conventional multi-port approach during thoracoscopic lobectomy.

## Introduction

Video-assisted thoracoscopic surgery (VATS) has been gained the wide acceptances on major lung resection as curative resection and is widely used in most centers [1, 2]. Most studies also have shown that the quality of life of patients who undergo surgery with this procedure is better than that of those who undergo thoracotomy [3–5].

Recently, single-port incision, or uniportal approach in VATS for lung disease has been reported as an attractive option for thoracoscopic surgery [6, 7]. Several groups who adopted the single-port VATS (SPVATS) have demonstrated acceptable oncologic outcomes and feasibility in major thoracoscopic procedures for lung malignancy [8]. Additionally, the greatest potential benefit of SPVATS is a better postoperative result regarding long-term pain, which is not resolved even with conventional VATS compared to thoracotomy despite a reduction in the number of ports [9].

We had reported our experiences of single-port VATS [10] and including two-port [11] surgery for major lung resection [12]. SPVATS is usually performed with a single 3 to 4-cm length skin incision without rib spreading. This procedure is expected to show similar or even better outcomes compared to the conventional multi-port VATS if technically feasible for the thoracic surgeon [13]. However, the adoption of this approach among them has not increased rapidly due to skepticism regarding the technical difficulty and increased operative risk based on the surgeon’s experiences; there have been great concerns regarding long-term oncologic clearances compared to established outcomes of conventional multi-port VATS [14]. In addition, there have been few studies of the long-term operative outcomes in large series compared with those of conventional multi-port VATS.

The purpose of this study was to evaluate surgical outcomes of patients undergoing SPVATS for major lung resection. We reviewed our 10-year experiences of conventional multi-port VATS and 4-year experiences of SPVATS for major pulmonary lung resection to address the benefit of this procedures.

## Materials and methods

### Study design and patients’ selection

**Fig 1:**
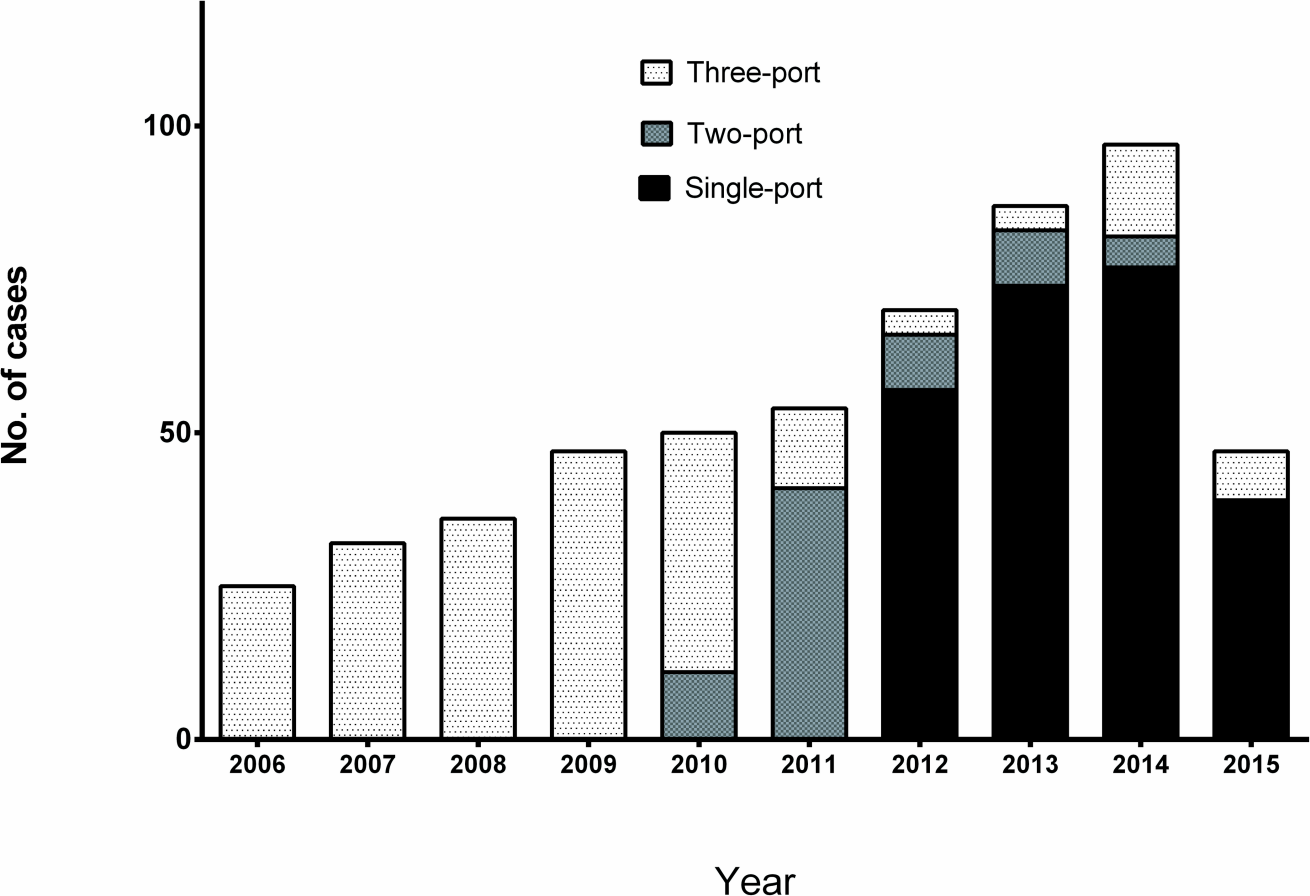
Changes of number of the port during thoracoscopic lobectomy.

We began VATS lobectomy in major lung resection in 2006 and launched SPVATS in patients with a benign disease in 2009, although the use of this approach was limited in simple minor procedures. Regarding major lung resection (more than segmentectomy) in patients with lung malignancy, following the learning period of two-port VATS lobectomy with more than 60 cases from 2010, we had changed our initial surgical approach for VATS lobectomy to a single-port approach since 2012 [15]. Patients who were selected for SPVATS for major lung resection according to our criteria were the same as those of conventional multi-port VATS lobectomy. Clinical stage I and II non-small cell lung cancer patients with acceptable operative risks were selected for this approach. However, cases of severe dense adhesion on preoperative CT scan were excluded from the initial indication of SPVATS and this single-port approach was optional based on intraoperative findings. The change of VATS technique in our institution showed on Fig 1.

The study included 247 patients who underwent SPVATS between November 2012 and June 2015. To compare the surgical outcomes of each VATS approach, we recruited data from previous cases of lobectomy with mediastinal lymph node dissection in NSCLC performed by VATS.

We collected the operative data including tumor size, number and extent of dissected lymph nodes, histology, pathologic stage, and follow-up data to determine the oncologic outcomes of SPVATS compared with conventional three- or two-port VATS. The first 50 cases of each VATS approach were selected to determine whether learning period of SPVATS was different from that of multi-port VATS approaches and to evaluate our strategy of two-port VATS experience for smooth transition to SPVATS lobectomy [16]. Our institutional review board approved and waived the individual consent and the retrospective data were collected excluding any personal information (IRB No KUGH13278-001) (S1 Appendix).

### Preoperative evaluation

Preoperative chest CT including the upper abdomen with additional bone scintigraphy where indicated was performed to exclude the hepatic and adrenal metastasis and whole-body PET scans were performed in all patients to exclude the extra-thoracic metastasis. The selected patients who showed lymph node enlargement or SUV uptake on mediastinum underwent EBUS or intraoperative cervical mediastinoscopy to exclude N2 disease.

### Operative procedure

The basic principles of the surgical techniques of conventional three-port and two-port procedures were described in previous studies [11]. Details of our technique for SPVATS lobectomy were as follows [10, 15]; one surgeon performed all surgeries with the same operative technique and approach throughout the study duration. Our routine position began with the lateral decubitus with a 3 to 4-cm incision at the fifth intercostal space on the anterior or posterior axillary line, according to the tumor location. In our cases, the surgeon always stands at the right side of the patient and the camera assistants standing on the left side regardless of the type of operation. There are several references regarding surgeon and assistant’s positioning to allow for a proper procedure without discomfort based on their own experiences. A wound protector was applied to achieve better instrumental performance, and to prevent contamination from cancer cells.

We routinely performed lymph node dissection in all lymph node station (more than 10 lymph nodes at upper, lower mediastinal, subcarinal and hilar station). A 5-mm or 10-mm thoracoscope were used in our procedure. We also used a 10-mm thoracoscope with three-dimensional view for better depth perception of the thoracic anatomy during the early learning period. We also performed SPVATS lobectomy or segmentectomy by using 2-cm single port with 3.3-mm thoracoscope in 2014.

A 5-mm articulating endoscopic device, graspers, and flexible and/or curved-tip endostaplers were used. An energy device was used for tissue dissection or hemostasis for small caliber vascular branch. Short-length vascular endostaplers (30-mm) or vascular interlocking clips were used to divide the interlobar vessel, if indicated. A 16- or 20-Fr sized chest drain and continuous analgesic pump system were routinely inserted through the port between the intrapleural space covering the multi-level intercostal area to reduce postoperative intercostal pain.

### Postoperative outcome

Data regarding perioperative courses, pathologic results including lymph node evaluation, and postoperative follow-up (survival and disease status) data were entered prospectively into our registry. The operation time was defined as the time from skin incision to closure. We defined the learning period of VATS lobectomy based on more than 50 surgical experiences for comparing the skill acquisition in the beginner [16]. Morbidity was defined as complications that required a reoperation, prolonged air leak (>5 days), and additional chest drains as well as postoperative event occurrence (<30 days) including cardiopulmonary problems (myocardial infarction, atrial fibrillation, pulmonary embolism, severe forms of pneumonia, respiratory failure, and acute respiratory failure) were classified according to the modified Clavien-Dindo classification [17]. The overall survival was defined as the time between the date of operation and that of death of any cause. The recurrence-free survival was defined as the time between the date of operation and that of cancer recurrence.

### Statistical methods

Summary statistics including demographic and outcome variables, including age, gender, location of tumor, types of operation, tumor size, the number of dissected lymph nodes, histologic cell type, pathologic stage, chest drain indwelling days after operation, postoperative morbidity, and postoperative follow-up periods were evaluated using chi-square tests for categorical variables and one-way analysis of variance (ANOVA) for continuous variables. Median follow-up time was calculated with reverse Kaplan-Meier method. Survival and recurrence-free survival curves were generated using the Kaplan-Meier methods, and the differences between curves were tested by the log-rank test using SPSS 23.0 (IBM Corp., Armonk, NY, USA). A p-values of less than 0.05 was considered significant.

## Results

### Patient characteristics

Since 2006, we performed three- and two-port VATS lobectomy in 154 and 58 patients, respectively, and SPVATS in 203 patients with non-small cell lung cancer between January 2012 and June 2015. Our SPVATS procedure included lobectomy in 167 patients, segmentectomy in 18 patients, extended resection in 10 patients, and wedge resection in 8 patients with non-small lung cell cancer (Table 1).

**Table 1:** VATS procedures for non-small cell lung cancer performed by type of the number of port.

	Three-port	Two-port	Single-port	P value
	(n = 168)	(n = 68)	(n = 203)	
**Age (mean, range)**	64.1 (40–86)	63.2 (44–86)	62.9 (33–84)	0.512
**Gender**				0.63
Male	105 (62.5%)	41 (60.3%)	132 (65%)	
Female	63 (37.5%)	27 (39.7%)	71 (35%)	
**Tumor location**				0.532
Right upper lobe	44 (26.2%)	26 (38.2%)	54 (26.6%)	
Right Middle lobe	17 (10.1%)	5 (7.4%)	22 (10.8%)	
Right lower lobe	34 (20.2%)	12 (17.6%)	48 (23.6%)	
Left upper lobe	35 (20.8%)	11 (16.2%)	45 (22.2%)	
Left lower lobe	38 (22.6%)	14 (20.6%)	34 (16.7%)	
**Operation**				0.009
Lobectomy	154 (91.7%)	58 (85.3%)	167 (82.3%)	
Segmentectomy	3 (1.8%)	3 (4.4%)	18 (8.9%)	
More than Lobectomy (bilobectomy, sleeve resection, pneumonectomy)	9 (5.3%)	7 (10.3%)	10 (4.9%)	
Wedge resection	2 (1.2%)	0 (0%)	8 (3.9%)	
**Conversion to thoracotomy or multi-port**	12 (7.1%)	3 (4.4%)	11 (5.4%)	0.911

Values are mean (range), n (%)

### Transition from three to single-port VATS

**Fig 2:**
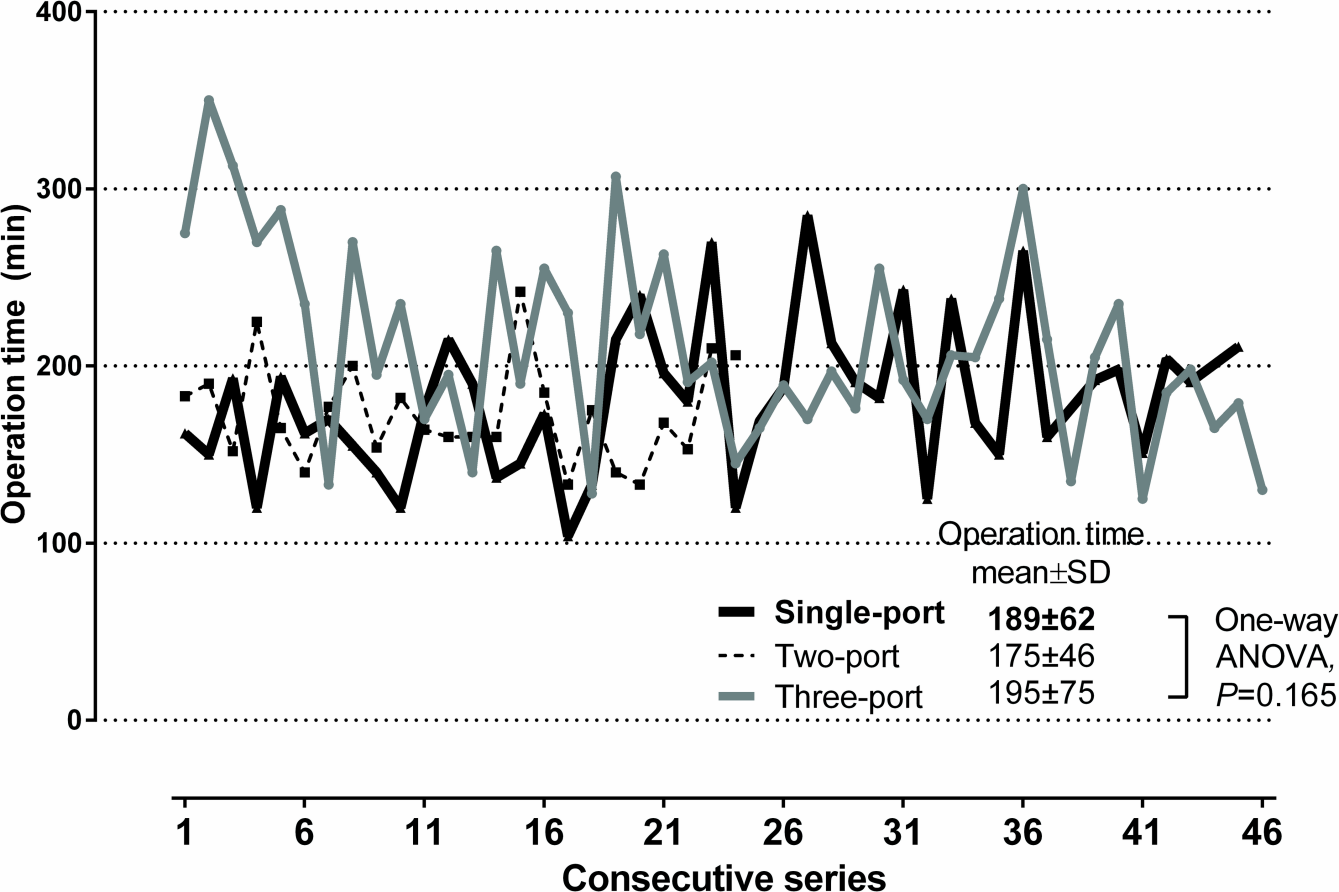
Operation times by the number of port during learning period of VATS lobectomy. SD = standard deviation.

Eleven cases were converted to mini-thoracotomy (n = 9) or multi-port VATS (n = 2) during the early period of SPVATS due to intraoperative bleeding (n = 6) or severe dense adhesion (n = 5). The conversion rate was not significantly different with that of three- or two-port VATS (p = 0.911). We analyzed the surgical outcomes of 167 patients who underwent SPVATS lobectomy with complete mediastinal lymph node dissection in patients with NSCLC confined to single lobe. During the learning period for each VATS approach, the overall mean operation times between each VATS approach was not significantly different (p = 0.165) (Fig 2).

**Fig 3:**
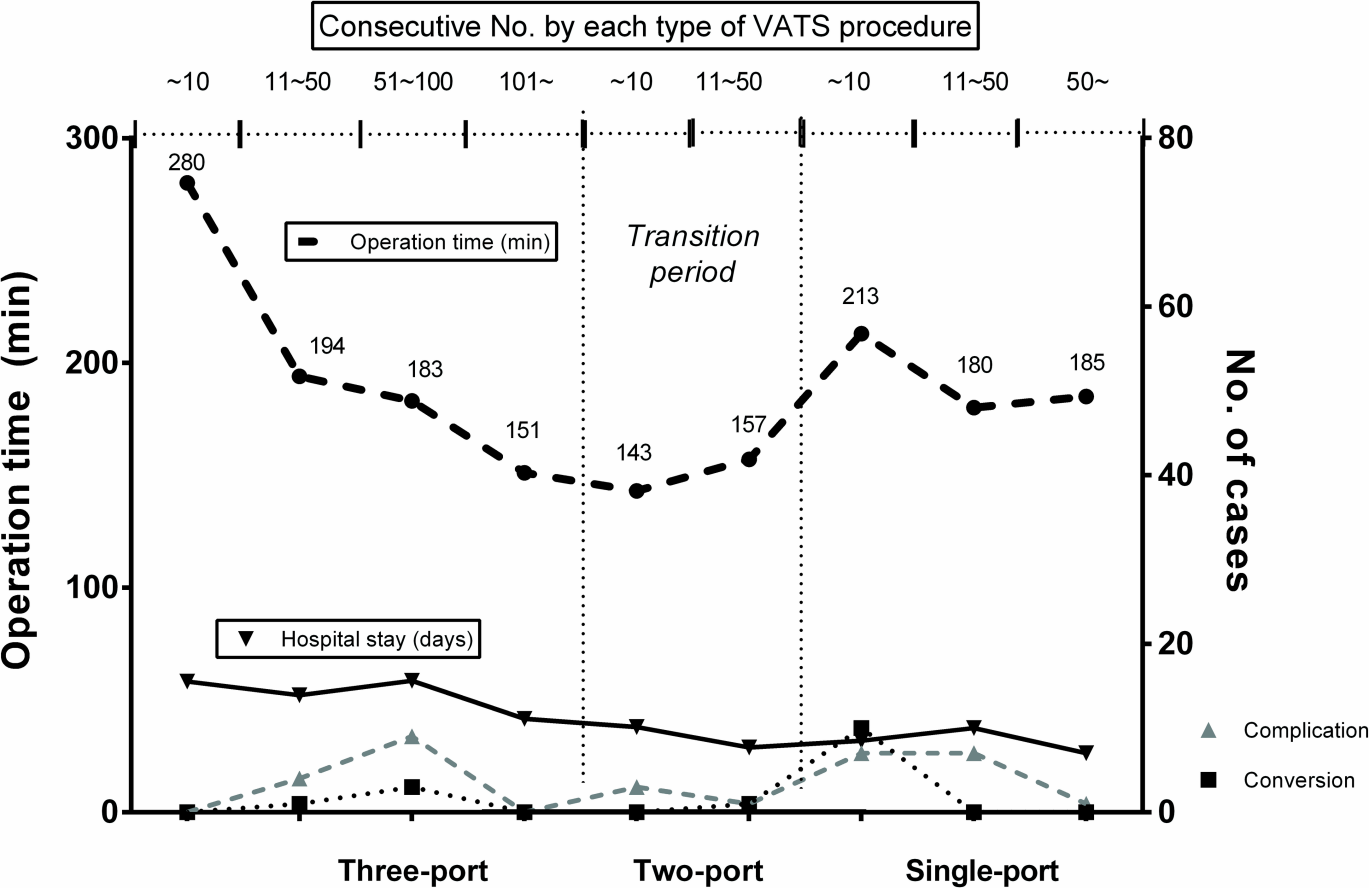
Operative parameters including operation time, hospital stay, complications and conversion to multi-port or open thoracotomy during transition from three-port to single port thoracoscopic lobectomy for non-small cell lung cancer.

The operation time for single-port thoracoscopic surgery was mean 189±62 (range, 104–285) minutes, which was not significantly different with that of two-port VATS lobectomy (175±46 [range, 133–242] minutes) (*p* = 0.779). However, the operation times of these two approaches were shorter than that of the conventional three-port VATS approach (three-port vs. two-port; p = 0.004, and three-port vs. single-port; p = 0.007). During early learning period of three-port VATS from open thoracotomy, the operation time was longer than those of two-port or SPVATS lobectomy. The changes of perioperative outcomes including hospital stay, postoperative complications, and conversion to multi-port VATS or open surgery are also shown in Fig 3. During the transition period of two-port VATS technique to SPVATS lobectomy, surgical outcome was not worsened.

### Outcomes

Parameters such as operation time, hospital stay, postoperative complications, and conversion events were improved as the surgeon gained experience. Tumor size (*p* = 0.475), the number of dissected lymph node (*p* = 0.512), histology (*p* = 0.085), and pathologic stage (*p* = 0.630) was not different between VATS lobectomy groups as classified by the number of port. Operative outcomes are summarized in Table 2.

**Table 2:** Operative outcomes by the number of port following VATS lobectomy for non-small cell lung cancer.

	Three-port (n = 154)	Two-port (n = 58)	Single-port (n = 167)	P value
**Tumor size (cm) Mean ± SD**	2.8 ± 1.7	2.6 ± 1.5	2.7 ± 1.1	0.475
**No. of dissected lymph nodes**	18 ± 11 (11–56)	20 ± 11 (8–52)	18 ± 9 (6–46)	0.512
**Histology**				0.085
Adenocarcinoma	86 (55.8%)	46 (79.3%)	113 (67.7%)	
Squamous cell carcinoma	49 (31.8%)	9 (15.5%)	46 (27.5%)	
Others	19 (12.4%)	3 (5.2%)	8 (4.8%)	
**Pathologic stage**				0.630
IA	66 (42.9%)	11 (19%)	75 (44.9%)	
IB	32 (20.8%)	19 (32.8%)	45 (26.9%)	
IIA	25 (16.2%)	13 (22.4%)	19 (11.4%)	
IIB	8 (5.2%)	10 (17.2%)	14 (8.4%)	
more than III	23 (14.9%)	5 (8.6%)	14 (8.4%)	
**Chest drain indwelling time (days),** **Mean ± SD (range)**	5.4 ± 2.1 (3–15)	4.3 ± 1.8 (3–12)	3.9 ± 2.2 (2–10)	<0.001
**Morbidity**	17 (11%)	2 (3.4%)	11 (6.6%)	0.185
**Median follow-up period (months),** **median (range)**	75.7 (1–124)	56.5 (4–69)	27.5 (1–53)	<0.001

SD = standard deviation.

**Fig 4:**
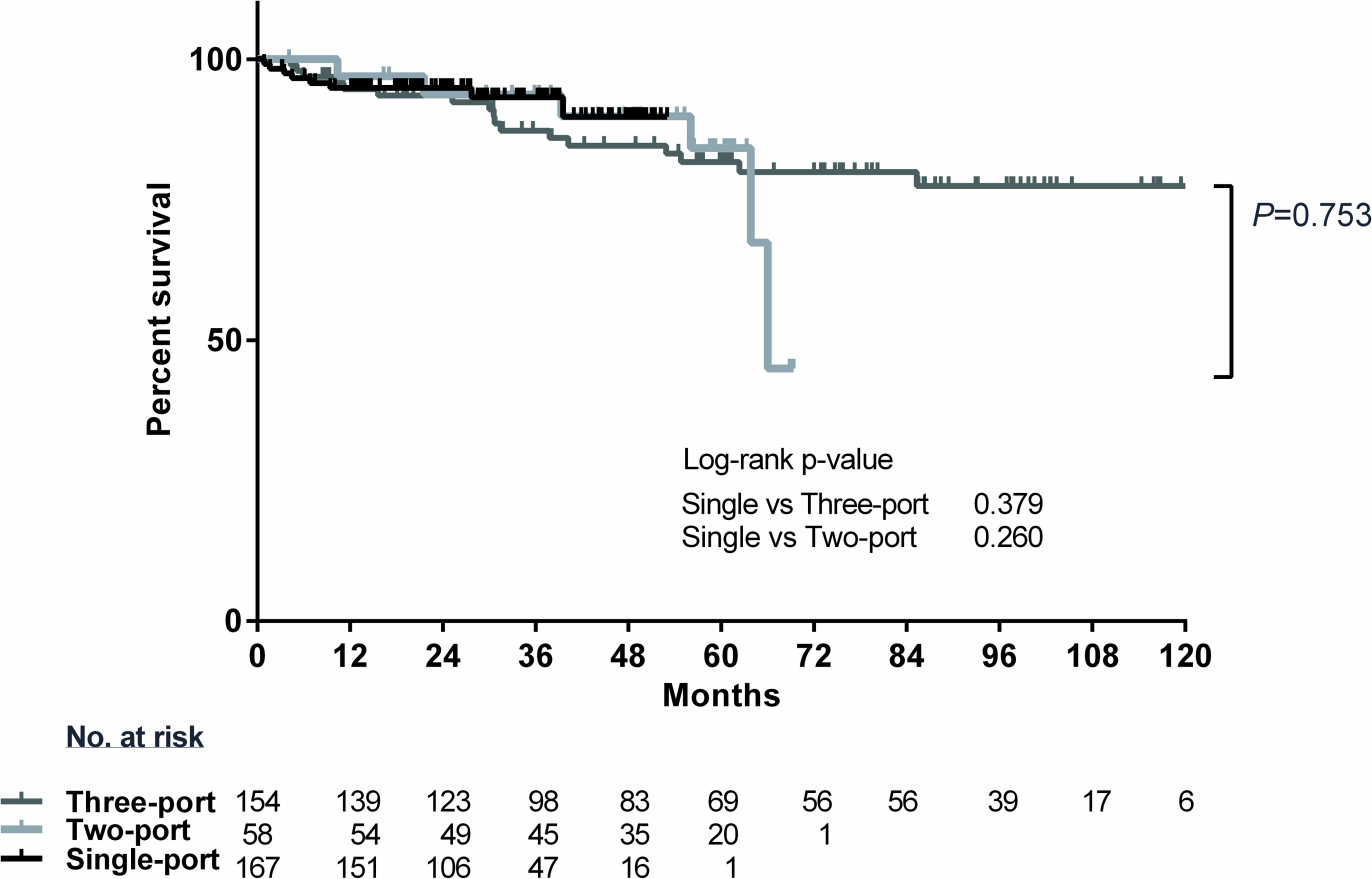
Survival by the number of port following VATS lobectomy for stage I non-small cell lung cancer.

**Fig 5:**
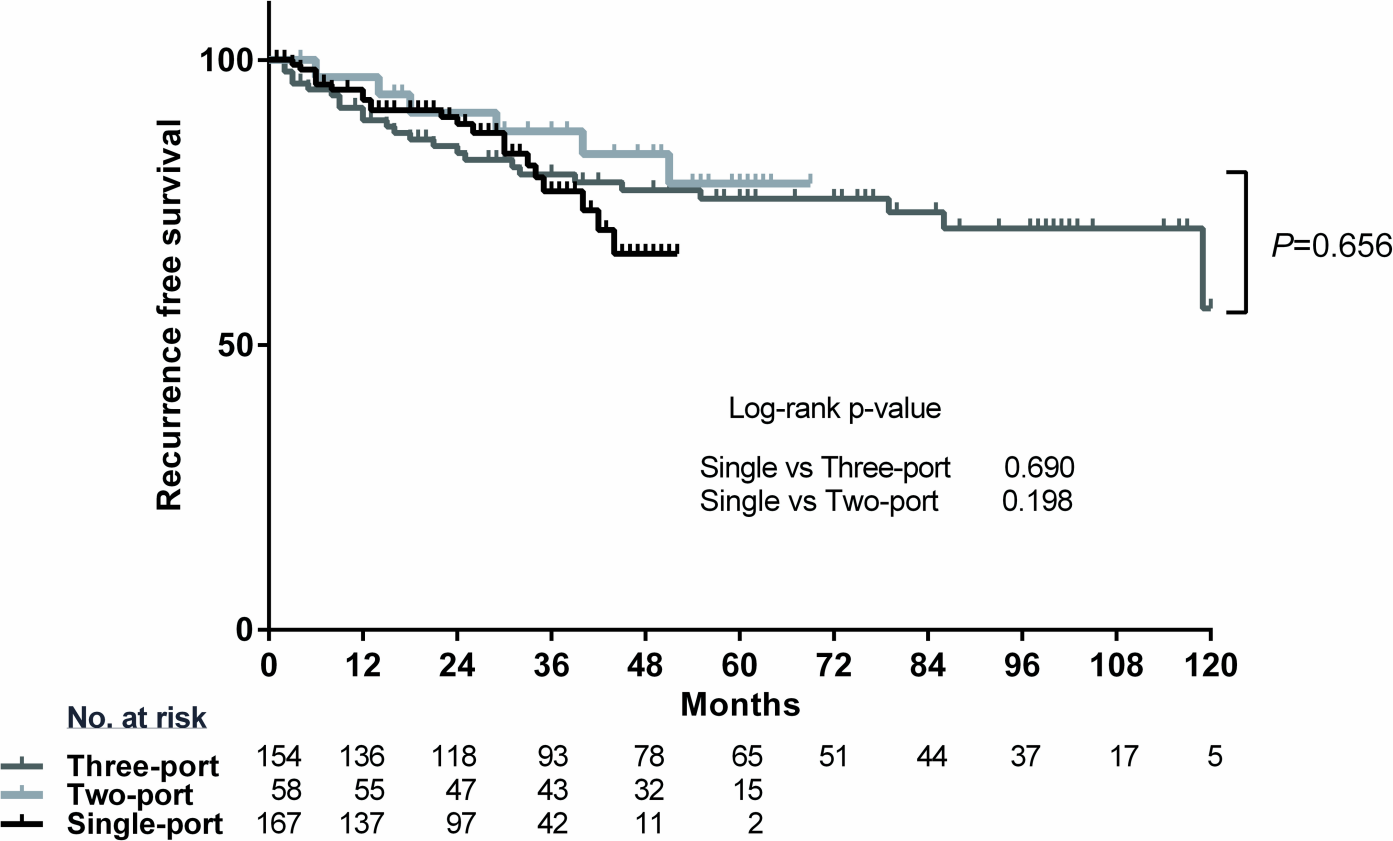
Recurrence free survival by the number of port following VATS lobectomy in stage I non-small cell lung cancer.

The median follow-up period was 27.5 (1–53) months in SPVATS, 56.5 (4–69) months in two-port VATS, and 75.7 (1–124) months in three-port VATS group. The survival curve of pathological stage IA and IB was not statistically different between the various VATS groups by number of ports (log-rank p = 0.753). The 3-year survival was 93.2% (95% CI, 85.7% to 96.8%) in SPVATS groups, 93.7% (95% CI, 77.2% to 98.4%) for two-port VATS, and 87.3% (95% CI, 78.1% to 92.8%) for three-port VATS (Fig 4). The recurrence-free survival at 3-years was 76.9% (95% CI, 64.6% to 85.5%) for SPVATS, 87.5%(95% CI, 69.9% to 95.1%) for two-port VATS, and 79.9% (95% CI, 69.9% to 86.9%) for three-port VATS. the difference was not significant between the approach (log-rank p = 0.656) (Fig 5).

## Discussion

Since the introduction of the single-port VATS approach, multiple groups have described the adoption of the single-port approach for VATS for various thoracic diseases [7, 18]. They reported technical feasibility and the safety of this elaborate strategy, although still considered technically difficulty for most thoracic surgeons. In the early period, pioneers of SPVATS had focused on the usefulness of SPVATS for simple thoracic procedures as this concept demands technical consideration during the manipulating of the instrument and surgeon’s patience, which was not problematic in cases of the simple thoracic procedure [19, 20]. Thus, they introduced this strategy cautiously as an alternative for the VATS-only procedure in simple thoracic surgery. This concept has not gained great interest and far from the thoracic surgical issue.

However, in the most recent 6 years, this concept was revisited and attracted attention as a feasible surgical approach for major lung resection and was indicated as an oncologically effective method for lung cancer surgery. Groups who performed SPVATS have showed that this approach is also a feasible approach to perform extended (bilobectomy or pneumonectomy) complex thoracic procedures including bronchovascular procedures [21], reconstructive sleeve procedures [22], or segmentectomy [23]. A few studies have reported on the oncologic efficiency of the single-port VATS approach for lung cancer surgery; it is, at least, not worsen compared with the result of the conventional multi-port VATS [13]. Our results also show acceptable outcomes in lung cancer surgery regardless of type of lung resection and acceptable for the extent and number of lymph node dissection. However, long-term results of more than 5-years are not currently available, because the current short follow-up period does not allow for this [24]. Our results during the intermediate term of SPVATS lobectomy in stage I non-small cell lung cancer showed acceptable outcomes compared to those of the multi-port VATS approach. Until now, several SPVATS groups reported comparable survival outcomes, but only in a relatively small case series using a variety of procedures during a short-term follow-up period [8]. In general, our results support the view that although survival following lobectomy is mainly stage-dependent, currently, it may be particularly effective for early-stage cancer (stage I).

The technical issue is a great concern for surgeons who consider single-port VATS for major lung resection. Such difficulty is a great hurdle to overcome, even for the active VATS surgeon [14]. To reduce the number of ports in the VATS approach, the surgeon might encounter an unfamiliar operative field, as it is different from that of the multi-port VATS, but might be similar to the thoracotomy view. In addition, surgeons also found limited instrumentation and a difficult angle for dissecting the vessel or stapling with the instruments for conventional multi-port VATS. Therefore, if a surgeon has little experience with multi-port VATS, they might encounter a catastrophic surgical event [25], such as intraoperative bleeding, resulting in conversion to open thoracotomy.

Our experiences of two-port VATS lobectomy during the transition period showed similar results to that of conventional multi-port VATS lobectomy without a worsening of the perioperative outcomes (operation times, complication, and conversion to additional port). To ensure the safe transition from the conventional three-port approach to SPVATS, we performed more than 100 two-port VATS procedure including more than 60 cases of two-port VATS lobectomy in patients with lung cancer by reducing the port number. In addition, during the two-port VATS period, we used the same endoscopic devices for conventional three-port VATS without the need for specially designed devices or a curving endoscopic camera in SPVATS [10]. We could avoid major changes of port placement of that of three-port VATS.

Groups who performed SPVATS had reported several methods to reduce the operative error and the time spent in the learning period. Currently, there is no consensus or best training method regarding the safe transition to SPVATS and reducing catastrophic operative events. It might depend on the surgeon’s surgical experience in conventional multi-port VATS; a surgeon must undergo many cases as their training strategy for SPVATS including simple cases to complex procedures in a step-by-step manner. Our transition strategy could be economically advantageous for training as it uses endoscopic devices and cameras for conventional multi-port VATS and might be helpful for young surgeons to reduce the errors during the learning period compared with that of direct transition to SPVATS.

The early period of the VATS approach has been criticized in terms of operative safety and outcome compared with open thoracotomy [26, 27]. However, currently, VATS has been developed to be on par with established standard thoracic procedures for better postoperative outcomes and has shown comparable or even better oncologic results. Additionally, similar to SPVATS, we should evaluate the acceptable results as the previous VATS trials have proven. Importantly, although many groups have reported better postoperative intercostal pain during the short-term follow-up interval (1–3 months) [28] or immediate postoperative period (< 7 days) and potential benefit of perioperative outcomes (smaller drain amount, shorter hospital stay) [7, 8], there has been no definitive evidence or clinical report on long-term intercostal pain in SPVATS of which a leading exponent has previously been described. Superiority of SPVATS over conventional multi-port VATS in terms of reduced intercostal pain is still controversial [9]. However, some experts suggested several tips for better instrumentation and operative view for SPVATS compared with multi-port VATS. In terms of operative view, the single port approach enables the endoscopic devices to move along a projectile plane that preserve the depth of visualization allowing bimanual instrumentation similar to open thoracotomy. In addition, SPVATS can prevent the torsional or dihedral angle with monitors created when multi-port VATS is performed [29].

Future study should clarify these questions on long-term intercostal pain, including the technical issues and operative safety, to include SPVATS as a standard in thoracic procedures.

## Conclusions

In summary, single-port thoracoscopic approach is safe for major lung resection and lobectomy in lung malignancy. Our experiences of SPVATS in single institution indicate that two-port VATS experience during transition period might allow the surgeon to perform a safe operation with acceptable operative outcomes.

## Supporting information

S1 Appendix (XLSX)
